# A New Vaccination Report

**Published:** 1913-07-12

**Authors:** 


### The Hospital

The Workers' Newspaper of Administrative Medicine and Institutional Life, Administration, National Insurance and Health.

No. 1411, Vol. LIV. SATURDAY, JULY 12, 1913. PRICE ONE PENNY.

### A NEW VACCINATION REPORT.

Our contemporary the *Lancet* has done good service in drawing attention to the latest report of an authoritative Commission of inquiry into vaccination for the prevention of smallpox, which might otherwise have escaped notice in this country. Two years ago the State of Pennsylvania appointed a Commission with a very wide scope of investigation into matters connected with smallpox, including, naturally enough, the whole subject of vaccination. The Commission comprised seven members: two eminent medical men, professedly favourable to vaccination; two anti-vaccinationists, respectively President and Secretary of the Anti-Vaccination League of America; and three neutrals, not medical men. The two medical members and two of the three neutrals signed a report the substance of which shall be presently set forth. The third neutral member produced a separate report, which is less enthusiastic about vaccination than that of the majority of the Commission, but is at least not anti-vaccinationist in tone. The two anti-vaccinationists signed a dissenting report which shows that they were not convinced by the evidence which satisfied the majority.

Before reciting the signed summary of the majority report, it is worth while to note the declaration appended to that report by the two neutral members, one a barrister and the other an insurance manager. They entered, according to their own statement, upon the performance of their duties with open minds, wholly free from any preconceived ideas or opinions upon the subjects of the reference; and they are convinced that the efficacy of vaccination and its relative harmlessness are so clearly established by the evidence that, in their judgment, not only is no other conclusion possible than as set forth in the report, but it would be difficult more strongly and conclusively to prove any fact of medicine or other science.

What are these conclusions, so firmly established to the satisfaction of two of the three unprejudiced members of the Commission? A survey of them discloses little that is favourable to the claims of anti-vaccinationists. Let us quote the words of the report. "The protective power of vaccination againt smallpox has been conclusively established. Successful vaccination protects against smallpox for a period averaging seven to ten years. Protection may be renewed on the exhaustion of the immunity by a second vaccination. Persons twice successfully vaccinated are usually immune against smallpox for life. Persons vaccinated in infancy, but not revaccinated, on contracting smallpox in later life have in the aggregate less severe and fatal attacks than unvaccinated persons."

This is a confident pronouncement as to the merits of vaccination as a preventive of smallpox, but not more so than the facts of experience in this country warrant. The next paragraph of the Pennsylvania investigators shows what vaccination does not do. "Vaccination," they proceed, "is a relatively harmless procedure, serious vaccinal injuries constituting but a minute fractional percentage and being nearly all preventible. Vaccination is safer now than ever before. . . . The number of alleged vaccinal injuries has been greatly over-estimated. There is no available substitute for vaccination in the prevention and suppression of smallpox epidemics. . . . "

Finally, as the document quite correctly observes, "this report is in harmony with the reports of all the official and Governmental Commissions that have considered this subject." Now the number of such official Commissions in this and other countries is considerable; and this uniformity of conclusion after complete investigation is so significant that anyone not blinded by prejudice and fanaticism would be able to appreciate it.

The evidence which satisfied the Commissioners of these findings we have not space to quote here. Suffice it that statistics and information from Europe and Britain were considered, as well as the experience of the United States. Of the Commissioners themselves, the two medical men were Professors Welch, of Johns Hopkins University, and Schamberg, of Philadelphia. The former of the two is the better known over here, where his name is a synonym for scientific eminence and honesty; but doubtless his colleague's record is almost equally good. The point is that neither of these distinguished men has a cent to make, directly or indirectly, by buttressing up the repute of vaccination falsely or fraudulently. If either of them were in the least doubt as to the correctness of any of the conclusions to which they have set their hands, neither of them would have consented to sign it. By training they are better qualified to judge on the matter than any of the other members, and this is no derogation from the intelligence and the experience of the neutral members of the Commission. Professors Welch and Schamberg are accustomed in their regular work to approach new problems with minds entirely free from bias; such detachment is the necessary state of any man who would do research of any value, and is pre-eminently to be found among University professors of scientific subjects. Thus the two medical representatives on the Commission were in no sense mere propagandists, deaf to all argument and blind to all demonstration, but men whose object is to attain knowledge of the truth, and whose training is designed to fit them for that quest. Such men are not found in the ranks of anti-vaccination—in this country at all events—and we may well doubt whether the two dissenting Commissioners were as well fitted to pronounce judgment on the issues referred to them. Anti-vaccinationists start out, in so many cases, with preconceived and fixed ideas about the "loathsomeness" of inoculating "animal matter" and similar vague question-begging phrases that they render themselves incapable of looking facts in the face. Accordingly, every few years it becomes necessary for some authoritative body to do this for them. The Pennsylvania Commission is the latest example; and we trust that it may help to spread the light of truth in this country, as well as in America. It will be interesting to see how the anti-vaccination exponents receive this serious shock to their creed.

